# Freud’s father religion: refinding *Moses and Monotheism* in 2023

**DOI:** 10.3389/fpsyg.2025.1706646

**Published:** 2026-01-09

**Authors:** David Titelman

**Affiliations:** Department of Learning, Informatics, Management and Ethics, National Center for Suicide Research and Prevention, Karolinska Institutet, Stockholm, Sweden

**Keywords:** antisemitism, Freud’s legacy, intellectuality, Jakob Freud, Judaism, *Moses and Monotheism*, October 7, 2023, physician-assisted death

## Abstract

At the inauguration of the Sigmund Freud chair of psychology at the Hebrew University in Jerusalem 1977, Anna Freud said that the rejection of psychoanalysis as a Jewish science “today can be regarded a title of honor.” The historian E. Roudinesco later discarded this claim as a “racist appropriation,” contrasting it with Edward Said’s allegedly anti-colonial reading of Freud’s major work on Judaism, *Moses and Monotheism.* Said’s in truth affectionate embrace of Freud’s *Moses*, however, influenced my openness to the book after October 7, 2023, and inspired the psychoanalytic re-reading of it that is the gist of this paper. Freud’s vacillation in publishing *Moses* in 1939 is scrutinized, as is his life-long combined professed godlessness and proud identification as a Jew. The discussion covers Freud’s interactions with colleagues and other interlocutors, which influenced his views on religion and antisemitism, and the extensive secondary literature on these and related issues. The ensuing understanding of Freud’s *Moses* is that it represents a self-analytic *tour de force* in which his father Jakob Freud, who until his death remained piously attached to traditional Judaism while also open to the Jewish Enlightenment, stands out as a profoundly impactful presence in Freud’s soul as well as in his legacy to the world.

## Introduction

Preparing a talk on Freud’s Judaism in the early fall of 2023, I – strange at it may sound – wanted to steer away from *Moses and Monotheism*, Freud’s major work on Judaism. This book, I thought, remains too controversial: it continues to be criticized within and outside psychoanalysis as poorly edited (e.g., Grubrich-Simitis, 1997) or, from a Jewish point of view, historically false or misguided (Baron, 1939; Kaplan, 2018; Julius, 2025). Yet, rereading it in the last minute before my talk (it was now November), I found it profoundly moving. I was not sure why, beyond the link between the emotions elicited by the attack on Israel that fall and the pervasive annihilation anxiety of 1939, when *Moses* was first published.

Freud at this time was 83, operated on for the 30*^th^* time for cancer in the palate, and forced to flee the Nazi invaders, who were escalating their campaign against the Jews of Europe to physical extermination. As if reproducing itself as a milder version of Freud’s need to speak out after having withheld the material of the third, concluding essay of *Moses* for more than a decade – it had, he wrote (1939a, p. 103), haunted him “like an unlaid ghost” – his book overpowered me and became a cornerstone of my talk in 2023 as well as in two subsequent presentations^1^.

While the overriding aim of this study remains to elucidate Freud’s Judaism, two specific questions evolved from the experience. The first question – why Freud’s *Moses* is so challenging to read – I have already answered, in part: the book’s shortcomings may stem from its having been completed under extreme existential stress. An additional strain may have been that the book’s multiple themes were sensitive: Freud wanted to summarize his lifework and legacy before it was too late; present a methodological framework for exploring his hypothesis on the Egyptian origin of Moses and his religion; and understand the riddle of antisemitism, if in no other way, from the point of view of what traits of the Jews themselves might provoke other people’s hatred. He did all of this by inviting the reader to follow the development of his numerous hypotheses and qualifications – substantiated or not by Egyptological, biblical, and archeological research as well as by clinical observations transposed to mass psychology. His digressions typically culminate in his re-introducing a previously questioned hypothesis either in the context at hand or in the book’s concluding essay, “Moses, his people, and the monotheistic religion,” which in Freud’s opinion was the centerpiece of his book and a supplement to the theory of religion that he had presented 25 years earlier in *Totem and Taboo* (Freud, 1914b).

Freud’s hesitation to publish his continued psychohistory of religion – now with an explicit focus on Judaism – was longstanding. In a letter written in 1934 to Arnold Zweig, a writer of historical novels who at the time lived in Palestine, he described an early version of his new text as a “novel (that) won’t stand up to my own criticism…, (a) statue upon feet of clay…that any fool could topple” (Ernst Freud, 1970, pp. 97–98). When he at last published this text as the third essay of *Moses*, Freud asked his readers to accept his reconstruction of the *historical truth* of what he had to say – a rendering of the psychological significance of the Moses story – and provisionally dispense with the empirical *material truth* sought by most academic historians.

While Freud’s interpretation of Moses was carried out in earnest, there is also self-irony in his formulations, presumedly directed against his own scientific frivolousness. May not comments as those just cited (“erecting a statue on feet of clay,” etc.), which were humorously shared with Zweig, also convey a graver, self-castigating dynamic, related to the predicament of both authors as German-language Jewish writers in 1934? The turning of aggressivity against the self among victims of life-threatening persecution is a recurring theme in the deliberations to follow^2^. Suffice it to mention now that it was impossible for both Freud and Zweig to publish anything in Austria or Germany at the time of this exchange; their earlier works had been publicly burnt there a year earlier (Wikipedia, 2008). Freud, further, declined Zweig’s offer to help him publish his “novel” (the third essay) in Jerusalem. He let this manuscript rest for the time being and eventually included it in *Moses*, which was published in Amsterdam in 1939, after he had resettled in London.

Answering my second question – what in this book moves some of us so profoundly – is the focus of what follows in this review. My method, call it a psychoanalytic reading (Titelman, 2006; cf., also, Green, 1986), includes a close reading of Freud’s writings on Judaism, a tacit reflection on my emotional response to what he wrote, and a review of a selection of the extensive secondary literature that his writings on Judaism has generated. There is no claim to present original historical material; the historical facts to be discussed have been established by earlier writers (e.g., Bergmann, 1982; Enckell, 2008; Grubrich-Simitis, 1997; Krüll, 1986; Rice, 1990; Robert, 1976; Schur, 1972; Shengold, 1972; Whitebook, 2017; Yerushalmi, 1991). What follows is rather, on a personal note, an attempt to recount my wrestling with Freud’s wrestling with his fate as a Jew.

## Anna Freud in Jerusalem

At the International Psychoanalytic Congress in Jerusalem 1977, the Sigmund Freud chair of psychoanalysis was inaugurated at the Hebrew University, on whose first board of governors, 1925, Freud had been a member together with Albert Einstein, Martin Buber, and the biochemist Chaim Weizmann (who was later to be appointed the first president of the state of Israel)^3^. Anna Freud, who was ill and unable to travel to Jerusalem at the time, had prepared an inaugural lecture that was read aloud in her absence. The following words concluded her lecture:

During the era of its existence, psychoanalysis has entered into connection with various academic institutions, not always with satisfactory results. It has also repeatedly experienced rejection by them, been criticized for its methods being imprecise, its findings not open to proof by experiment, for being unscientific, even for being a “Jewish science.” However the other derogatory comments may be evaluated, it is, I believe, the last-mentioned connotation which, under present circumstances, can serve as a title of honor (Freud, 1978, p. 148).

About 30 years later, in a book titled *Revisiting the Jewish question*, the historian Roudinesco (2013) devalued Anna Freud’s formulations in Jerusalem as an appropriation of the “abject expression…used by the Nazis to destroy the language and doctrine of psychoanalysis” (p. 91). Roudinesco further claimed that the inauguration took place in a restricted meeting, closed to the congress participants. (I *was* a congress participant and can testify that the event took place in the open). She then contrasted Anna Freud’s contribution with that of the Palestinian-American professor of literature, *Edward Said*, who in 2002 had given an allegedly radically different perspective on Freud’s identification as a Jew in a talk on Freud and Moses at the Freud Museum in London. (In all of this, I hear Roudinesco doing her utmost to disqualify Anna’s both sad and creative fate as a surviving refugee of the Holocaust as well as her strong attachment to her father and grief after his death^4^). That the venue of the intimated showdown was Freud’s and Anna’s last home, from their arrival in London in 1938 until Anna Freud’s s death in 1982, adds to the insolence of Roudinesco’s attack.

Reading Said’s lecture, which was later published by the museum (Said, 2003), I, however, found it relevant to my question about the impact of Freud’s *Moses* on its readers. Said begins by depicting the tragedy of the Palestinian people’s displacement and never-ending exclusion from the community of open societies of the West. His profound grief provides a steppingstone to Freud’s book: he views Moses, an assumed Egyptian, as a co-creator of a people and praises the boldness of Freud’s rendering of him as such as well as an object of his – and Said’s own – longing for a father. The affective tone of Said’s identification with Freud stands in stark contrast to Roudinesco’s criticism of Anna Freud and other distinguished Freudians.

## It’s hard to be a Jew: Freud, Jung, and Abraham

If we provisionally accept the notion of psychoanalysis as a Jewish science, we must also acknowledge that Freud emphasized that the fact that psychoanalysis was discovered by a Jew did not mean that it was more Jewish than, for example, Einstein’s theory of relativity. In 1907 he befriended *Carl Gustav Jung* whom he hoped would assume the role of future leader of the psychoanalytic movement and thereby thwart the widespread perception of psychoanalysis as a Jewish affair and promote its acceptance in academic psychiatry. Enthralled by Jung, Freud regarded his and the other Swiss psychiatrists’ interest in psychoanalysis as gift from heaven. Yet, early on, he noticed an anti-Jewish inclination in Jung, which perception he did his utmost to suppress. This attitude was particularly noticeable when Freud defended Jung against his skeptical colleagues in Vienna and Budapest and not the least *Karl Abraham*, a psychiatrist who had worked with Jung in the Burghölzli hospital in Zürich, since 1904. An example of Freud’s complacency is found in a response to Jung, who in a letter had complained about Abraham, whom Freud had not yet met; Jung accused Abraham of stealing his ideas. Freud responded empathetically and asked, as one haughty non-Jew might ask another: “By the way, is he a descendant of his eponym?” Jung replied that Abraham was in fact what his name implied (Freud and Jung, 1974, p. 80).

Was such subtle condescension genuinely anti-Jewish? Freud did occasionally express mixed feelings about things or attitudes Jewish, for example, certain religious practices and the unpolished manners of some Jews, including among his followers. My impression is, however, that Freud’s problem in this regard was a matter of class-consciousness, a personal social complex, as also reflected in his discovery in himself and others of self-idealizing “family romance” fantasies (Freud, 1909). This phenomenon, which in Freud’s view has Oedipal roots, was, as we shall see, also prominent in his understanding of Moses.

A common response to questions about Freud’s ostensibly mixed feelings about being Jewish is indeed to explain them as Oedipal (e.g., Gay, 1988; Robert, 1976). Freud (1900) himself embraced this perspective, for example, in the oft-cited episode from *The Interpretation* of Dreams in which he contrasted his childhood hero, the “semitic” Hannibal, whose warrior father *Hamilka Barca* (*Barak* in Hebrew) demanded that he strike back at the Roman enemy, to his own, mild-mannered father, Jakob Freud. Freud recalled that his father told him how he on a Saturday stroll in their hometown Freiberg in Moravia (today Příbor in the Czech Republic) was once assaulted by an antisemitic hooligan who knocked off his new fur hat – likely a Hassidic-style *streimel* (Bergmann, 1995) – and ordered him to get off the sidewalk. When the 10–12-year-old Sigismund, as he was originally named^5^, asked his father how he had responded to the attack, Jakob answered that he had done as he was told, stepped down from the sidewalk and picked up his hat. Bergmann noted that Jakob Freud’s restraint, which was censured by his stern son as cowardly, was in fact realistic: the Jews of Moravia at the time the hat-incident was likely to have occurred were forbidden by law to use the sidewalks. They were granted full civil rights only after 1867, when the Austro-Hungarian empire was established.

Resisting antisemitism continued to be challenge at the turn of the century, when politically motivated anti-Jewish discrimination re-emerged in Europe (Klein, 1985; Wasserstein, 2012). To undergo conversion to Catholicism or Protestantism for social reasons remained an option for the religiously indifferent Jew hoping to be socially included as a German or a Frenchman – or a Scandinavian – even when that choice was also a narcissistic defeat, intensifying feelings of guilt and loneliness. Chasseguet-Smirgel (1988) has written that it may have been more favorable for an individual’s self-regard to remain in the fold of the Jewish religion, whose promise of being chosen and a sense of belonging compensated for its prescribed renunciation of instinctual pleasure and idolatry.

Karl Abraham, who after meeting Freud became increasingly dedicated to the study of psychoanalysis, shared with him not only a passion for the new discipline but also an interest in Jewish history, including antisemitism, of which both men had first-hand experience. This relationship was nonetheless, on Freud’s part, cooler than his bond with Jung, which historians have described as a love affair with bouts of madness (Haynal and Falzeder, 2002; Whitebook, 2017). Jung, who was uncomfortable with Freud’s sexual theory as well as with his intolerance of challenges to his authority, was intermittently beset by Christian mystical visions. At one point he uncannily and, as noted by Whitebook (2017), naively (bearing in mind Freud’s theory of the Oedipus complex), suggested that the two men annul their differences and find peace by thinking of themselves as father and son, thereby positioning himself as a Christ-like successor. It is conceivable that Freud initially was open to such talk; he wanted to support Jung and was not uninterested in the historical Jesus. But mysticism, whatever its religious or ideological context, was incompatible with his outlook.

Freud sensed that Abraham was a suitable target for the antisemitic projections that the colleagues in Zürich did not dare direct at himself, and noted in a letter: “I think that we as Jews, if we wish to join in anywhere, must develop a bit of masochism.” (Freud, 1908b, p. 54). In the same vein he had earlier recommended that Abraham, facing Jung’s fallibilities, remain calm:

Be tolerant, and do not forget that really it is easier for you to follow my thoughts than it is for Jung, since…you are closer to my intellectual constitution through racial kinship, while he as a Christian and a pastor’s son finds his way to me only against great inner resistances. His association with us is therefore all the more valuable….It was only by his emergence on the scene that psychoanalysis was removed from the danger of becoming a Jewish national affair (Freud, 1908a, p. 38).

Abraham’s warnings about Jung’s antisemitic leanings were later to be crudely confirmed. In 1933 the professional guild *Allgemeine Ärztliche Gesellschaft für Psychoterapie* came under Nazi control and was renamed *Deutsche Allgemeine Ärtzliche Gesellschaft für Psychoterapie*. Under the guardianship of the psychiatrist Matthias Heinrich Göring – a cousin of Hermann Göring – “non-Aryan” members were expelled from the society, and a decree was issued that the work of its members henceforth be based on Hitler’s *Mein Kampf*. Ernst Kretschmer promptly resigned as the guild’s chairperson and was as promptly replaced by Jung, who also became editor of its journal (Jones, 1957).

## Hard to be a Jew…(continued)

The Vienna psychoanalytic society, originally the “Wednesday group,” which had convened in Freud’s home since 1902 and which Jung, Binswanger, and Abraham visited in 1907 (Abraham at a later occasion than the others), at the time had 17 regular members, all Jews. At the society’s final board meeting 1938 in which Richard Sterba, who was not Jewish, also participated^6^, it was agreed that all members who could find the means to do so should leave the country and that the seat of the society should be wherever Freud settled. Referring to the Talmud, Freud said:

After the destruction of the Temple in Jerusalem by Titus, Rabbi Yochanan ben Zakkai asked for permission to open a school in Yavneh for the study of the Torah. We shall do the same. We are after all used to persecution by our history, tradition and some of us by personal experience (Jones, 1957, p. 236).

Notwithstanding that these words convey Freud’s grounding in Jewish thought, his hope when he formed the Wednesday group had been that it would be a non-denominational replacement of his earlier arena, the Jewish philanthropic and politically liberal fraternal society *B’nai Brith Wien*. Between 1895 and 1902 this men’s club had been a haven to which he could retreat from the increasingly aggressive antisemitism of academic life in Vienna. The more than 10 lectures on early psychoanalytic hypotheses that he presented to B’nai Brith have been reconstructed by Klein (1985). For a society meeting held in 1926 to honor his 70th birthday, long after he had ceased to be an active member, Freud wrote an address that included the following words:

…In the years from 1885 and onwards…my unpleasing discoveries had as its result the severance of the greater part of my human contacts; I felt as if I were despised and universally shunned. In my loneliness I was seized with a longing to find a circle of…men of high character who would receive me in a friendly spirit in spite of my temerity. Your society was pointed out to me as the place where such men were to be found.
That you were Jews could only be agreeable to me; for I was myself a Jew, and it had always seemed to me not only unworthy but positively senseless to deny that fact. What bound me to Jewry was (I am ashamed to admit) neither faith nor national pride, for I have always been an unbeliever and was brought up without any religion though not without a respect for what are called the “ethical standards” of human civilization. Whenever I felt an inclination to national enthusiasm, I strove to suppress it as being harmful and wrong, alarmed by the examples of the peoples among whom we Jews live. But plenty of other things remained…to make the attraction of Jewry and Jews irresistible – many obscure emotional forces that were the more powerful the less they could be expressed in words, as well as a clear consciousness of inner identity, the safe privacy of a shared mental construction. And beyond this there was a perception that it was to my Jewish nature alone that I owed two characteristics that had become indispensable to me in the difficult course of my life. (As)…a Jew I found myself free from many prejudices which restricted others in the use of their intellect; and as a Jew I was prepared to join the Opposition and do without the agreement with the “compact majority” (a reference to Ibsen’s *Enemy of the People*) (Freud, 1926, pp. hbox273–274).

## Freud and Moses

### The Moses of Michelangelo

Despite his declared godlessness, Freud was profoundly interested in religion. Like Spinoza he saw it as a necessary consolation for most people, albeit that the ideal he upheld for himself and fostered in his patients was to confront reality without illusion. Throughout his life he also admired Moses, an affection that matured from an adolescent veneration of the prophet as a physically powerful hero into a later appreciation of his mental strength and integrity. In psychoanalytic terms this development can be understood as reflecting a late resolution of an unintegrated oedipal relationship with his father, an advance that took place only after Jakob Freud’s death in 1896, when Freud’s unanticipated grief impelled him to begin a systematic self-analysis.^7^

In 1901, on his first visit to Rome, where he had traveled with his younger brother Alexander, Freud for the first time saw Michelangelo’s statue of Moses holding the tablets of the law, located in the sixteenth-century church *San Pietro in Vincoli*. He was overcome by his hero’s imposing physique as well as the self-containment and capacity for shrewd thought and action that he felt that Michelangelo had captured in his sculpture. Freud knew very well that this perception reflected his *wish* that Moses had these traits and that neither historical research nor the Bible provided support for it. (In the biblical story, Moses, descending from Mount Sinai and confronting the people’s adoration of the golden calf, raged against their apostasy and shattered the tablets of the law).

It would take Freud thirteen years to complete his short study, *The Moses of Michelangelo* (Freud, 1914a), in which he presented his idealized vision of Moses. When the text was finally published, he insisted on anonymity – against the advice of his editors (Jones, 1955). Freud’s publication anxiety in this instance arouses one’s curiosity. Should we understand his Moses fantasy of 1914 as a daydream, an epiphany, a projection of his own controlled anger and containment, or perhaps as a displacement of his suppressed love for his father?

A widespread speculation is that the ongoing upheaval and conflicts in the psychoanalytic movement interfered with Freud’s writing at this time. He completed the Michelangelo manuscript only after another trip to Rome, accompanied by his sister-in-law Minna Bernays, with whom he traveled to recuperate after the international psychoanalytic conference in Munich 1913, the last such meeting before World War 1 and the occasion when Jung’s secession became official (Jones, 1955).

In 1913, however, Freud also had unfinished business with Karl Abraham, who was now an established psychoanalyst and respected leader of the Berlin Psychoanalytic Society. A year before the completion of Freud’s *Moses of Michelangelo*, Abraham – encouraged by Freud (Hilda Abraham, 1974) – published a study titled *Amenhotep IV (Ikhnaton): A psychoanalytic study of his personality* (Abraham, 1935). In this work he portrayed the Egyptian pharaoh *Amenhotep IV*, who was crowned when he was 10 years old and died at 28, and who during his short reign (1353-1336 BCE) created an astonishingly modern religion, a cult of the life-giving power of the sun represented as *Aten* (meaning solar disk in ancient Egyptian). Under the young king’s rule, the traditional worship of the god *Amon*, which was headed by his father *Amenhotep* III, was abolished. Religious practices other than rational contemplation on reality were forbidden, representations of the previously worshiped gods in the Egyptian temples were demolished, and new statues or pictures, including of the young king himself, were banned. Further, as the claimed offspring of the divine power (*Aten*), the younger Amenhotep changed his name to *Ekhnaten* (or *Ikhnaton*) – he was now the son of God – and eradicated all traces of his biological father from the historical annals.

Abraham noted Ikhnaton’s Oedipal traits, which (in addition to the above) were also reflected in his order that his mother *Tiy*, when she died, should not be lain to rest in her husband’s burial chamber, as stipulated by tradition, but in the chamber reserved for himself^8^. Tiy, whose title was Great Royal Wife, had been brought to Amenhotep III’s court to from “Asia^9^.” Her young age, beauty, and vigor, Abraham suggested, must have attracted her son and – together with her Eastern origin – contributed to the characteristics of the new religion. The abstract, all-powerful single deity, the taboo against making an image of him, and the religious founder’s imagined divine origin are recognizable ideas, respectively in Judaism and Christianity.

Freud advised Abraham not to label Ikhnaton as neurotic, noting that Oedipal traits can befall healthy and creative individuals too (Hilda Abraham, 1974). The point is fair, notwithstanding that the young king comes across as consistently grandiose and morally corrupt. It is stranger that Freud, as far known, did not acknowledge Abraham’s contributions either in *The Moses of Michelangelo* (1914a) or in *Moses and Monotheism* (1939). In both of these works Ikhnaton’s monotheism – which Abraham (1913, 1935) was the first to illuminate psychoanalytically in print – was at the center of Freud’s analysis^10^.

### Moses and Monotheism

When, after Ikhnaton’s death, his successors deferred to the priesthood and reintroduced polytheism, Moses remained loyal to the deceased king’s teachings. Oppressed by the renewed idolatry, he, in Freud’s (1939a) reconstruction, found a similarly marginalized people with whom he established an alliance: the Jews, who were grateful to be chosen. Freud understood the psychology of this elevation in terms of the earlier mentioned *family romance*: like Ikhnaton before them the Jews became God’s children, and the circumcision of their men, he wrote, was an unerasable sign of their chosenness. This claim, too, collides with the biblical story of Abraham (the patriarch) as the originator of the practice of circumcision as a mark the Jews’ covenant with God. Freud audaciously discarded this narrative as a legend, invented by the writers of the Bible to “lessen if not totally suppress the true significance of Moses” (pp. 44–45).

A critical addition in Freud’s analysis concerns Moses’s fate during the Jews’ generations-long flight out of Egypt. The story he tells, which again seems to differ from the biblical account, demands of the reader to accept certain ideas about the prehistory of our civilization, including the notion that there may have been two Jewish leaders named Moses:

Wandering in the desert, under the pressures of external harsh conditions as well as of the strict demands of the Mosaic laws, which, Freud suggests, were even more exacting than Ikhnaton’s code of behavior, the Jews revolted and murdered Moses, which deed he understands as a repetition of the prehistoric murder of the primal father (Freud, 1914b). This crime was forgotten but not completely so. It was encapsulated as an unconscious memory and a source of a feeling of guilt that was reactivated when the fleeing Jews eventually encountered another Jewish tribe in *Kadesh* in the Midianite (Arabian) desert. The all-powerful deity of this group of Jews, *Yahveh* (which name was to become forbidden to utter), was originally an awe-inspiring volcano-god who, like the God of Moses, was linked to a sacred mountain, “Sinai-Horeb” (different from Mount Sinai on the Sinai Peninsula). And in this story, too, a divinely inspired leader, also named Moses, descended from the mountain. Not only was the power of the Midianite god greater than that of the of the vanquished gods of the neighboring peoples, but his might was also more palpable than the abstract resourcefulness and moral demands of the god of the Egyptian Moses^11^. A compromise was negotiated by the two groups: the version of Judaism that exists to this day. The Midianites were concerned “only in denying the recency…of their god…and in heightening his claim to the people’s devotion” (Freud (1939b), p. 77), whereas the descendants of the followers of the original Moses …

would not renounce (the) memories of the liberation from Egypt and the magnificence of…the leader Moses; and, indeed…succeeded in finding place for the fact as well as for the man in the new representation of Jewish early history, in retaining at least the outer sign of the Moses religion – namely, circumcision – and in insisting on certain restrictions in the use of the new divine name” (p. 76).

As mentioned, Freud considered the three essays of *Moses and Monotheism* a summary of his intellectual legacy to the world. The psychoanalytic hypotheses he made use of this time around (every major publication by Freud includes a renewed discussion and refinement of the never-completed body of *metapsychology*, his theory of the unconscious) included perspectives on: repression and the return of the repressed; latency phenomena in memory and in the development of individuals as well as groups; his for decades withheld intuition of a phylogenetic dynamic – call it epigenetic – in individual development; self- and other-directed destructiveness and the counterforces against it; and the fateful splitting phenomena in individuals, groups, and nations that were increasingly manifest in external reality in Freuds’ last years.

At the international psychoanalytic congress in Paris 1938 – the last such meeting before World War II – which the precarity of his situation in Vienna prevented him from attending, Freud selected a part of his Moses manuscript for Anna to read to the delegates. His choice was a chapter from its third essay, titled *Der Fortschritt in der Geistigkeit* or, in Strachey’s translation, *The advance in intellectuality* (Freud, 1939a)^12^. Against the background of the large-scale destructiveness of the times, Freud’s appeal to reason and creativity calls for further elaboration. In addition to the circumstances already mentioned (Freud’s age, illness, personal losses, and his transformation into an exiled refugee), one must consider that the explicit goal of the Nazi occupiers of Vienna was to destroy his lifework (Roudinesco, 2016). Primo Levi was later to describe this aspect of Nazism, as it was played out in Auschwitz, as a wish to kill the Jews’ souls before exterminating their bodies (Titelman, 2006).

Grubrich-Simitis (1997) understood the impact of the Nazi invasion of Freud’s mind as working in tandem with his fear of losing his autonomy – a vulnerability that she saw as grounded in never-analyzed pre-oedipal, maternal areas of his internal world. She suggested that his choice to publish *Moses* in 1939 despite its “compositional disharmony” (p. 57) was his way of curing himself from the injuries the Nazis were in the process of inflicting on him, and that he psychically survived by reaching safety in London and reconquering the ability to work before he died. Grubrich-Simitis labeled the deficiencies of *Moses*, particularly those of its third essay, as “scar tissue” (p. 79) in need of further working through and healing. Be this as it may, is there not an intense self-analytic train of thought just beneath the surface in this part of *Moses*, for example, in Freud’s discussion of the development of religion: from totemism and the omnipotence of thought – and the lesser known matriarchal order that Freud acknowledges may have preceded totemism – to the view of guilt-driven man found in Judaism and Christianity?

The direction of…this father-religion was…laid down for all time….Ambivalence is a part of the essence of the relation to the father: in the course of time the hostility of the father could not fail to stir….(But) there was no place in the ….religion of Moses for a direct expression of the hatred of the father (nor for the repressed memory of the prehistoric murder of the primal father, whose law, according to Freud, was symbolically reinstated by Moses). All that could come to light was a mighty reaction against it – a sense of guilt on account of that hostility, a bad conscience for having sinned against God and for not ceasing to sin. This sense of guilt, which was uninterruptedly kept awake by the Prophets…soon [after the compromise in Kadesh] formed an essential part of the religious system (Freud, 1939a, p. 134).

Freud continued this reflection on how history has contributed to shaping the Jewish character:

The [Jewish] people met with hard times; the hopes based on the favor of God were slow in being fulfilled; it became not easy to adhere to the illusion, cherished above all else, that they were God’s chosen people. If they wished to keep their happiness, then the consciousness of guilt…offered a welcome excuse for God’s severity. They deserved nothing better than to be punished by him, because they did not observe the laws; the need for satisfying this feeling of guilt, which, coming from a much deeper source, was insatiable, made them render their religious precepts…ever more strict, more exacting, but also more petty. In a new transport of moral asceticism, the Jews imposed on themselves constantly increasing instinctual renunciation and thereby reached – at least in doctrine and precepts – ethical heights that had remained inaccessible to the other peoples of antiquity (Freud, 1939b, p. 173, trans. K. Jones).

Further, Freud notes that Saul (who would become Saint Paul), a Jewish type with whom he was familiar, was driven by his need for punishment to breakdown, only to rise again with a redefined religious outlook: Christianity. In this new, universal religion, the guilt feelings of the Jews after the forgotten patricide were transformed into original sin the dysphoric affect of which was alleviated by the external drama of the sacrifice of Jesus; chosenness was replaced by salvation. For the Jews, the price for this revolution was high. The new Christians, who in their hearts remained polytheists, would in the times to follow accuse the Jews of being murderers of God and of denying their guilt. “It was as if Egypt had come back to wreak her vengeance on the heirs of Ikhnaton,” Freud (1939b, p. 175) wrote.

As to the reception of *Moses*, Freud, who was deeply worried about the reaction of the Catholic church (the state church of Austria), seemed not to be overly anxious about the Jewish response to his book, even though he in a letter asked the designated Hebrew translator of *Moses* “to consider that its contents might hurt Jewish sensitivity in so far as there is a reluctance to submit the subject to scientific inquiry” (Rice, 1990, p. 46). This formulation comes across as insensitive to the more likely objection of most Jews to hearing, in their darkest hour, that their greatest prophet was not a Jew but an Egyptian, never mind that Freud, ever the revealer of hidden identities, seemed to take pride in Moses being an aristocrat or a close relative of the pharaoh.

Did Freud overestimate the willingness of the Jews to follow him in his psychoanalytic mind games? Not only did he feel safe among Jews, his confidence rested on a base of healthy narcissism one source of which was the life-long love he received from his proud father, the onetime *Hassid* turned *Maskil*, which in Jakob Freud’s case meant a pious man who, while open to the *Haskalah* (the Jewish Enlightenment) and to budding modernity, remained steadfastly attached to Jewish tradition, including the study of the Talmud and the holy language. Beyond Jakob’s loyalty, however, Freud’s wish that his readers should follow him in his work on Moses without reservations seemed to be asking too much. This was so even though his main point in the book was *not* – this deserves to be repeated – to capture the materially true history of Moses but the psychological leap in the history of civilization, from a dependence on sensory experience to the advance of intellectuality, reflection, and – I write this with a nod to Edward Said – wistfulness.

## Jakob Freud’s legacy

In this reading, *Moses and Monotheism* stands out not as Freud’s rejection of Judaism but, on the contrary, as a testimony to his re-alignment with it, not the least with the piety and sagacity of his father. Solid support for Jakob Freud’s subtle presence in Freud’s writings in general and monumental return in *Moses* is provided by Yosef Hayim Yerushalmi, professor of history, ordained rabbi, and clearly at home in the symbolic universe of Freudian psychoanalysis. His *Freud and Moses:* Judaism Terminable and Interminable (1991) includes a transliteration and translation of the hand-written, cursive-script dedication that Jakob Freud wrote Hebrew in the bilingual (Hebrew-German) Bible that father and son had studied together in Freud’s childhood. In 1891, when Freud turned 35, Jakob gave him this family treasure, which he had rebound in leather, as a birthday gift.

The dedication is written in the form of *melitzah*, a compilation of internally resonating citations from the Bible and the rabbinic literature. This literary device was employed in Hebrew poetry and prose from medieval times to the Haskalah and the 19*^th^* century into our own times by traditional as well as more modern Jewish writers. The lines in such a text have literal as well as associative meanings, which of course are best understood if you are knowledgeable in scripture, which Freud was to a greater extent than he tended to admit in public (Bergmann, 1982; Rice, 1990; Yerushalmi, 1991). Some specimen citations:

The very first words of the dedication, “My son Shlomo” (*ben-yakir li Shlomo*) – Shlomo was Freud’s Hebrew name – derive from Jeremiah 31:19, “Truly, Ephraim is dear to me” (*ha ben yakir-li Efrayim*). And the last words of the dedication, “who loves you with ever-lasting love” (*ahavkha ve ahavat olam*), are from Jeremiah 31:9, “with ever-lasting love have I loved thee” (*ve ahavat olam ahavtikha*). In Jeremiah, Yerushalmi writes, the beloved son, Ephraim, is “a symbol for those wandering in exile, which “is coupled with a promise of return and reconciliation with God the father” (p. 73).

Jakob Freud continued by praising his son who “heard and strove to do,” paraphrasing Exodus, 24:7, “All that the Lord has spoken we will do and hear,” and as a result has “soared on the wings of the Spirit,” which refers to the book of Psalms 18:11, “He soared upon the wings of the wind” (…*al kanfi haruach*) – the Hebrew word *ruach* denoting both soul and wind. “Since the book was abandoned,” Jakob Freud wrote, it has been “stored like fragments of the Tablets in an ark [*aron*],” signifying the Ark of the Covenant between God and Moses. “I have put upon it a cover of new skin,” and the ensuing citation express Jakob’s hope that his restored gift may inspire a return to its study and be a source of renewal: “Spring up O well. Sing ye onto it” – these are words from the *Song of the Well* in the Book of Numbers 17:11 in which water miraculously rose for the Israelites wandering in the desert (Yerushalmi, 1991, p. 73)^13^.

Yerushalmi underscores that the idea that the broken tablets are restored through piety and study – lost wisdom, like material shards, can be repaired – is not from the Bible but the commentaries of the Talmud. He also makes a well-grounded psychoanalytic interpretation that Jakob Freud’s wish for his son’s return stayed with Freud for the rest of his life. It was this reconciliation that Freud, when his world was being shattered, conveyed in *Moses.* And it was this quality of his book that moved me upon rereading it in the fall of 2023.

## Epilogue

Freud died in 1939 in London, after his physician, Max Schur, who had followed him from Vienna, honored their agreement 11 years earlier to end Freud’s pain when that was all there was. On September 21, Freud, barely able to talk, indicated to Schur that this time had arrived. Schur informed Anna and the next day administered two low- but lethal-dose injections of morphine over a 12-h period. Freud died the following morning, September 23 (Schur, 1972). This day was Yom Kippur, the Jewish Day of Atonement, when the communal prayers focus on death, remembrance, and spiritual rebirth.

As he wished, Freud – contrary to Jewish tradition – was cremated three days later. His ashes were placed in a South-Italian, Apulian bell krater with a Dionysiac motif (Turner, 2007), which Freud had received as a gift from Marie Bonaparte in 1931, when he turned seventy-five. In his letter of thanks, he wrote her: “…a pity that I cannot bring it with me in the grave” (Schur, 1972, p. 560, my trans. (original citation in German)). For unknown reasons the urn, which was placed on a pedestal designed by one of Freud’s sons and situated in a columbarium in Golders Green in London, was vandalized in 2014. Renovated, it can be seen today behind a protective screen. We need not know how Freud himself would have reacted to this unusual monument. We can merely assume that he would at least have smiled at it. Freud’s outlook was life-affirming, not without humor, and there was certainly a corner in his soul that was open to hedonistic pleasure.

In the third essay of *Moses*, in which man’s destructiveness as well as its necessary counterforces – love of self and Other, and Oedipal realism – are elaborated with a minimum of technical jargon, Freud wrote that we don’t really need to reconstruct a materially true experience of The Jews having murdered Moses as a primal father or God. Their guilt feelings are understandable without such a myth: human ambivalence and internal conflict suffice to explain the anxieties, including guilt feelings (the underlying aggressivity of which sometimes joins forces with hate from others) and are intrinsic to life. Conflict and mental pain need to be dealt with intelligently by the struggling individual, not magically disposed of. Freud concluded “The advance in intellectuality” (his words of farewell to his psychoanalytic followers in 1938) with the following words. I read them as cautionary:

The pre-eminence given to intellectual labors throughout some two thousand years in the life of the Jewish people has, of course, had its effect. It has helped to check the brutality and the tendency to violence which are apt to appear where the development of muscular strength is the popular ideal. Harmony in the cultivation of intellectual and physical activity, such as was achieved by the Greek people, was denied to the Jews. In this dichotomy their decision was at least in favor of the worthier alternative (Freud, 1939a, p. 115).

## Footnotes

1 The presentations were given at the conference *Limmud* Stockholm, November 2023, The Nordic Psychoanalytic Congress in Oslo, August 2024, and the symposium, “Freud and Judaism,” organized by *Judisk kultur i Sverige* (*Jewish Culture in Sweden*), April 2025.

2 The anguish of survivors of such assault and the need to find ways to heal their mental injury appear in the discussions of or references to Freud, Anna Freud, Primo Levi, Edward Said, and Arnold Zweig.

3 In 1929 Freud was also appointed honorary member of the presidium of YIVO, the Yiddish Scientific Institute of Vilna (Vilnius), Lithuania (Yerushalmi, 1991). (YIVO relocated to New York in 1940.)

4 Anna Freud, whose four aunts (Freud’s sisters) perished in Theresienstadt and Treblinka, 1942, was arrested and interrogated by the Gestapo in Vienna before her and her father’s escape. She was released only after the intervention by the US Embassy and possibly by Eduardo Weiss, a psychoanalyst in Rome who is said to have mobilized Mussolini’s support to free her. Freud’s benefactor, Marie Bonaparte (the great-grandniece of Napoleon Bonaparte), waited at his side at home for Anna’s return (Edmundson, 2007; Sigmund Freud Museum, 2025).

5 The name of the religiously tolerant, sixteenth-century King Sigismund III (Sigismund Vasa) was popular among Galician Jews. In Vienna, however, it was a target of antisemitic ridicule, wherefore Freud, upon entering *Gymnasium* in 1865, registered his name in its German form, *Sigmund* (Klein, 1985; Rice, 1990).

6 Sterba, who was deeply attached to Freud, left Vienna two days after the meeting (Jones, 1957; Sterba, 1961). As a founding member of the Detroit Psychoanalytic Society, he in 1940 continued to show both originality and integrity: the society was the first psychoanalytic institute in the USA to welcome nonmedical candidates, which led to Sterba’s temporary disbarment as a training analyst by the American Psychoanalytic Association (Meisels, 2016); his observations of the split between the experiencing and observing ego, the latter of which allies itself with the analyst (Sterba, 1934), prefigured the concept of a *therapeutic alliance*, which concept was also dogmatically contested in America in the 1950s. Sterba died in 1989.

7 In a letter after his father’s death to his early confidant Wilhelm Fliess, Freud (1896) wrote: “Dear Wilhelm, I find it so difficult to write just now that I have put off for a long time thanking you for your warm words in your letter. By one of those dark pathways behind the official consciousness, the old man’s death has affected me deeply. I valued him highly, understood him very well, and with his peculiar mixture of deep wisdom and fantastic light-heartedness he had a significant effect on my life. By the time he died, his life had long been over, but in my inner self the whole past has been reawakened by this event. I now feel quite uprooted.” (p. 202, Trans. J. M. Masson).

8 DNA tests on material collected 2010 have confirmed that the remnants of Tiy are preserved in different royal burial chambers in Egypt between which they appear to have been moved in different time periods (Mark, 2011).

9 Baron (1939) noted that the pharaoh Amenhotep III’s wives were brought from Syria.

10 Freud’s omission has been analyzed in detail by Shengold (1972).

11 In the Bible, Moses flees to Midian after witnessing an Egyptian beating a Hebrew slave, killing the offender, and hiding the corpse [Freud does mention this incident in a separate discussion of Moses’s (1939a, p. 32)] personality. Before his escape, Moses encounters two men brawling with each other, realizes that his crime is known to others, and admonishes the assumed instigator of the brawl, who then asks: “Who set you up as a *man* prince and judge over us?” (Exodus 2:4, trans. R. Alter, italics added). Without addressing Freud directly, Alter (2019) provided two notes to his translation of th Hebrew Bible that are relevant to our discussion: (1) “These words of the brawler in the wrong not only preface the revelation that Moses’s killing of the Egyptian is no secret but also adumbrates a long series of later incidents in which the Israelites will express resentment and rebelliousness against Moses. Again, ‘man’ is stressed. Later ‘the man Moses’ [which expression Freud also uses throughout his book] will become an epithet for Israel’s first leader” (p. 217); (2) “The geographical location of Midian in different biblical references…[is] not entirely fixed, possibly because the Midianites were nomads. Moses’s country of refuge appears to be a semidesert region bordering Egypt to the East, to the west by northwest of present-day Eilat” (p. 218).

12 Kathrine Jones translated *Geistichkeit* as spirituality (Freud, 1939b).

13 There are other English translations of the dedication. The earliest one is penned as a draft onto the pages of the family Bible itself by Freud’s English- and to a sufficient degree Hebrew-speaking, elder half-brother, Emanuel. Making use of this draft, Rice (1990) produced a new professional translation and used it as a point of departure of *his* psychoanalytic book on Freud and Moses (published a year before Yerushalmi’s). Yet another translation was published by Ostow (1989). Aside from differing exegetic and psychoanalytic commentaries by the respective writers (Ostow, Rice, and Yerushalmi), their translations are largely in harmony with one another. Ostow, however, underscored that three themes run through Jakob Freud’s citations: love, hard mental work, and the ensuing reward of the flow of “reason, knowledge, and understanding” (Ostow, 1989, pp. 486–487).
